# Enhancement of *Cunninghamella elegans* UCP/WFCC 0542 Biomass and Chitosan with Amino Acid Supply

**DOI:** 10.3390/molecules180910095

**Published:** 2013-08-22

**Authors:** Ednaldo Ramos dos Santos, Marta Cristina Freitas da Silva, Patrícia Mendes de Souza, Antonio Cardoso da Silva, Sergio Carvalho de Paiva, Clarissa D. C. Albuquerque, Aline E. Nascimento, Kaoru Okada, Galba M. Campos-Takaki

**Affiliations:** 1 Biological Sciences Program, Federal University of Pernambuco, Recife 50670-901, PE, Brazil; E-Mail: santosmestrado@gmail.com; 2 Nucleus of Research in Environmental Sciences, General Coordination of Research, Catholic University of Pernambuco, Recife 50050-590, PE, Brazil; E-Mails: tyttams@hotmail.com (P.M.S.); albqqs@yahoo.com.br (C.D.C.A.); elesbao@unicap.br (A.E.N.); kao@unicap.br (K.O.); galba_takaki@yahoo.com.br (G.M.C.T.); 3 Center of Biodiversity Studies, Federal University of Roraima, 69309-270 Boa Vista, RR, Brazil; E-Mails: martacfs@yahoo.com.br; 4 National Program of Post-doctorate (CAPES), Catholic University of Pernambuco, Recife 50.050-900, PE, Brazil; 5 Department of Education, State of Pernambuco, 50.810-000 Recife, PE, Brazil; E-Mail: antoniocardoso2000@yahoo.com.br; 6 Center of Sciences and Technology, University of Pernambuco, Recife 50050-900, PE, Brazil; E-Mail: spaiva@unicap.br

**Keywords:** *Cunninghamella elegans*, chitosan, sucrose, asparagine, corn steep liquor

## Abstract

Studies were carried out with *Cunninghamella elegans* UCP/WFCC 0542 to evaluate the effects of an abundant supply of amino acids, asparagine and corn steep liquor associated with sucrose on the production of biomass and chitosan by submerged fermentation. The concentrations of the components of the culture medium which were determined by a 2^3^ full factorial design evaluated the interactions and effects of the independent variables of the sucrose, asparagine and corn steep liquor in relation to carbon and nitrogen sources, on the production of chitosan regarding biomass. The best results were observed at the central point [asparagine 0.025%, sucrose 0.15% and 0.45% of corn steep liquor, ratio C:N=2:6], and produced maximum yields of 16.95 g/L biomass and 2.14 g/L chitosan, after 96 h of submerged fermentation. However, the lowest level of sucrose, asparagine and corn steep liquor produced a low amount of biomass (10.83 g/L) and chitosan (0.60g/L). The infrared spectrum absorption of the chitosan produced by *C. elegans* showed bands regarding OH-axial stretching between 3406 and 3432 cm^−1^, superimposed on the NH stretching band with axial deformation of the amide C=O group at about 1639 cm^−1^, NH angular deformation at approximately 1560 cm^−1^; axial deformation of amide-CN at around 1421 cm^−1^, symmetrical angular deformation in CH_3_ at 1379 cm^−1^, -CN axial deformation of amino groups from 1125 to 1250 cm^−1^ and polysaccharide structure bands in the range of between 890–1150 cm^−1^. The crystallinity index of chitosan was 60.92%, and its degree of deacetylation was 75.25%. A low percentage of a supply of sucrose and asparagine with corn steep liquor offered higher yields of biomass and chitosan production at low cost.

## 1. Introduction

The fungus *Cunninghamella elegans* UCP/WFCC 0542 was isolated and characterized in our laboratory. It can produce chitin and chitosan [1,2], catabolize xenobiotic compounds, especially azo dyes [3,4,5] and heavy metals by biosorption [6], in addition to which it can perform the biological desulfurization of organic sulfur dibenzothiophene—DBT [7], which the literature on *C. elegans* has corroborated [8].

The nutrients used for microbial growth depend on a certain balance of carbon and nitrogen sources which is needed for the secondary production of metabolites. In order to use industrial waste, knowledge of the quantity and quality of carbohydrates, proteins or lipids to be used as substrate for producing biotechnological compounds is needed. Thus, agro-industrial substrates which can be used as renewable sources for an alternative technology that produces biomass and chitosan are sought. This requires the optimization of important parameters such as agitation speed, temperature, pH, and particularly, carbon and nitrogen sources [9,10,11,12,13,14,15,16,17].

Among the renewable sources obtained from agribusiness, corn steep liquor is undoubtedly one of the most widely used products. Corn steep liquor is a co-product from the wet milling of maize that is rich in amine compounds, such as essential amino acids, vitamins, and other nutrients and is used as a food supplement in the manufacture of feed for poultry and ruminants. However, corn steep liquor is also a low-cost nutritional source, which is effective as a nitrogen source in various fermentation processes for high value-added input [18,19,20].

Chitin is the second most abundant organic compound in nature, after cellulose. Chemically it is a long-chain unbranched polysaccharide made of *N*-acetylglucosamine residues. Structurally, chitosan is a straight-chain copolymer composed of a *N*-deacetylated derivative of chitin, although this *N*-deacetylation is almost never complete, D-glucosamine and *N*-acetyl-D-glucosamine being obtained by the partial deacetylation of chitin [21,22]. Chitin is found mainly as a component of fungal cell walls, parasitic nematodes, crustacean shells, yeast, and insects [23,24]. Some researchers consider chitin is a naturally occurring fibre-forming polymer that plays a protective role in many lower eukaryotes similar to that of cellulose in plants [25,26,27].

Chitin is obtained from the shells of various types of shellfish, including shrimp and crab; and chitosan, as the most abundant amino-polysaccharide can be obtained from chitin by a deacetylation process. Chitin and chitosan are considered biodegradable and biocompatible homopolysaccharides produced by natural sources, and versatile and promising biomaterials. The degree of deacetylation (DDA) of chitosan often has an important parameter related to many physicochemical and biological properties which include solubility, viscosity, polyelectrolyte behavior, polyoxysalt formation, the ability to form films, metal chelations, optical and structural characteristics, such as crystallinity, and hydrophilicity [28,29]. Chitosan is used in several fields such as medicine, pharmaceutical, agriculture, and food industries due to its versatility. However, studies are being carried out investigating potential problems with the consumption of chitosan from marine origin since some individuals are allergic to crustaceans and a relationship between allergy reactions and chitosan is presumed [30,31].

Considerable research work has been carried out on using mycelium waste from fermentation processes as a source of fungal chitin and chitosan, especially Mucoralean fungi, which has great biotechnological potential, given that the content of chitin and chitosan in their cell walls is significant [32,33,34,35,36,37].

The goal of this research paper was to obtain biomass and chitosan by *Cunninghamella elegans* UCP/WFCC 0542 using sucrose and asparagine at low concentrations as substrates, and a supply of natural amino acid with corn steep liquor. This paper mainly examines the inﬂuence of an abundant supply of amino acids on enhancing the production of biomass and chitosan by *C. elegans* UCP/WFCC 0542.

## 2. Results and Discussion

### 2.1. Effects of Sucrose, Corn Steep Liquor and Asparagine on the Production of Biomass and Chitosan

Initially an investigation was made of the nutritional requirements of *Cunninghamella elegans* for the components of the medium of sucrose, corn steep liquor and asparagine, given a C:N ratio of 2:6 (excess nitrogen source), on the production of biomass and chitosan, per the independent variables, as shown in Table 1. The results showed that supplying corn steep liquor increases the production of biomass, influenced by carbon/nitrogen ratio in all assays of the factorial design, except 1, 3, and 7, respectively, given the low level of the nitrogen source. The highest production of biomass and chitosan was observed at the central point of 2^3^ full factorial designs, at which the highest values were observed, these being 16.95 g/L for biomass and an excellent production (2.14 g/L) of chitosan.

The data obtained suggest that the association of sucrose, asparagine with corn steep liquor (asparagine 0.025% and 0.15% sucrose with 0.45% corn steep liquor) presents the best condition. The initial pH ranged from 4.01 to 4.66 and rose to 5.10 to 5.50. The pH of the medium changed but remained in the acidic range which suggests this is the optimum condition for the activity of the enzyme chitin deacetylase for chitosan production, given that the optimum enzyme pH is 4.5, according to Kafetzopoulos [38]. The increase of biomass is explained by enrichment brought about by amino acids (leucine, isoleucine, lysine, methionine, tyrosine, phenylalanine, threonine and serine), and vitamins (biotin, choline, inositol, niacin, pyridoxine, riboflavin, thiamine and pantothenic acid), which, according to Bento [39], are essential nutritive sources for producing biomass and chitosan by *C. elegans*.

**Table 1:** Biomass and chitosan produced by *Cunninghamella elegans* on sucrose, asparagine and corn steep liquor using 2^3^ full factorial design.

Assay	pH Initial	pH Final	Biomass (g/L)	Chitosan (g/L)
1	4.66	5.30	10.83	0.60
2	4.01	5.10	11.12	1.51
3	4.02	5.46	14.62	1.07
4	4.59	5.09	10.63	1.93
5	4.01	5.48	14.64	2.03
6	4.01	5.40	14.72	1.52
7	4.03	5.37	13.47	1.04
8	4.02	5.15	14.02	1.55
9	4.03	5.44	16.95	2.10
10	4.02	5.21	16.03	2.08
11	4.01	5.50	15.77	2.12
12	4.01	5.29	15.52	2.14

Experimental design 2³ for the production of biomass and chitosan: Asparagine %: −1 (0); 0 (0.025); 1 (0.05); Sucrose %: −1 (0.1); 0 (0.15); 1 (0,2) ; Corn Steep Liquor %: −1 (0.3); 0 (0.45); 1 (0.60).

Studies undertaken by Streit *et al*. [40] using *Gongronella butleri*, Mucoralean fungus, obtained 1.19 g/L of chitosan after 72 h of cultivation, while *C. elegans* produced a lower amount on using sucrose and asparagine supplemented with corn steep liquor. Cardoso *et al*. [20] using corn steep liquor and honey as medium components to produce biomass and chitosan by *Rhizopus arrhizus* found yields of 20.61 g/L and 29.3 mg/g respectively. However, the results showed that *R. arrhizus* produced a higher biomass but a lower amount of chitosan than those obtained in this study.

### 2.2. Assessment of Independent Variables on the Production of Biomass and Chitosan

**Figure 1:**
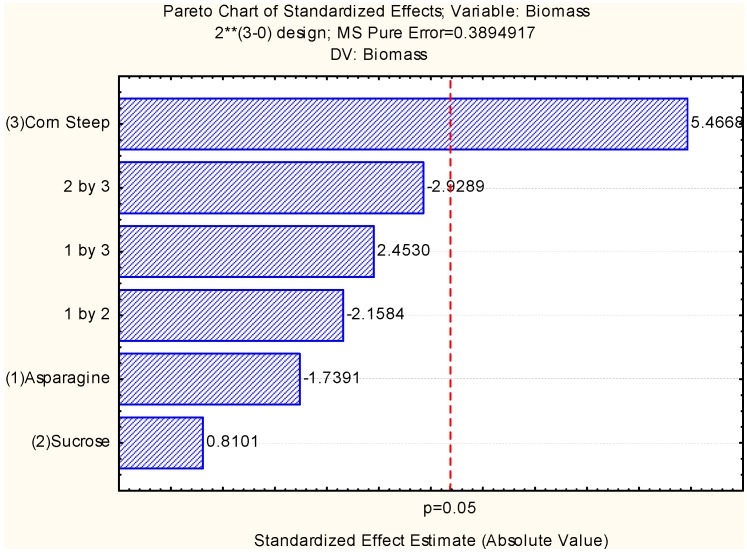
Pareto Chart of standardized effects for 2^3^ full factorial design when the independent variables are: (1) asparagine (2) sucrose (3) corn steep liquor, and (4) corn steep concentrations as a response variable on biomass production by *Cunninghamella elegans*.

The experimental data obtained in this study were subjected to statistical analysis to evaluate the effects of the components of the culture media (sucrose, corn steep liquor and asparagine) on the biomass yield. The Pareto chart showed the standard effects of a 95% confidence level with the factors of sucrose, asparagine, and corn steep liquor. The analysis of the factors and levels used demonstrate that the independent variable of only corn steep liquor was the most positive variable of the chemical components on the yield of biomass production (Figure 1). However, sucrose alone did not positively influence the growth of the microorganism, suggesting *C. elegans* prefers corn steep liquor. Therefore, the results suggested that there was a better physiological adaptation of *C. elegans* when corn steep liquor (agroindustrial residue rich in essential amino acids, vitamins and minerals) was present. These results are supported by the literature on the biomass production of filamentous fungi, yeast and bacteria using corn steep liquor as an essential source in the chemical composition of the culture media used in fermentation processes [7,8,20].

**Figure 2:**
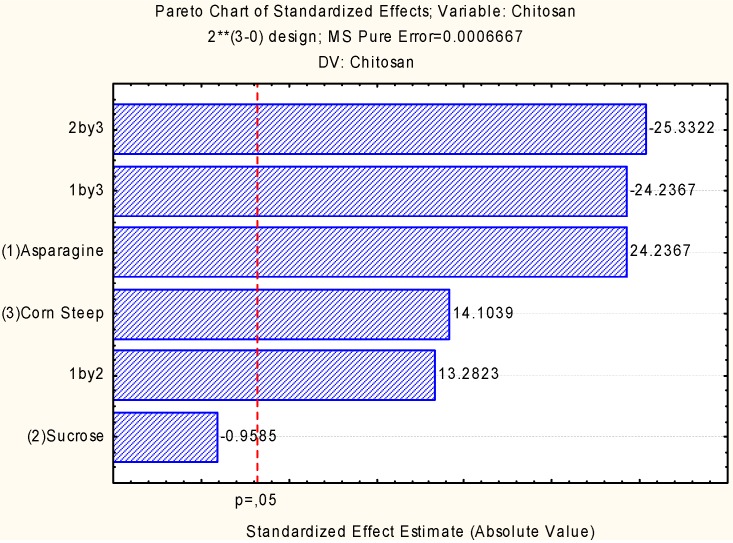
Pareto Chart of standardized effects for 2^3^ full factorial design having as independent variables: (1) asparagine (2) sucrose and (4) corn steep concentrations and as the response variable the chitosan yield by *Cunninghamella elegans* related to carbon and nitrogen sources.

Figure 2 gives a Pareto chart, the results of which show the influence of the nutritional sources on the production of chitosan in the interaction of the asparagine and corn steep liquor, and asparagines and sucrose on the formation of the biopolymer. The results also show the influence of asparagine and corn steep liquor on the increase in chitosan yields, that of sucrose and asparagine being statistically significant for the yields of chitosan by *C. elegans*. However, as to the interaction of sucrose and corn steep liquor, and the interaction of asparagine and corn steep liquor, negative effects were observed, with statistical significance on the chitosan yield. With a higher concentration of corn steep liquor, an agroindustrial residue, a rich supplement of amino acids was assimilated by the microorganism tested when chitosan was produced.

These results are supported by the literature as to not only the positive effect on the uptake of constituents of corn steep liquor, in amounts of 6% to 16% [18,19] but also as to the positive influence on the increased production of chitosan with supplementation of only 0.45% of corn steep liquor.

Figure 2 shows response to interactions between asparagine and corn steep liquor, asparagine and sucrose and corn steep liquor, and in detail the supplementation effect of corn steep liquor. The most positive influence on chitosan production was observed in the interaction between sucrose and corn steep liquor, and the high values of chitosan production by the fungus *Cunninghamella elegans* are shown.

The interaction between sucrose and asparagine was demonstrated to be a negative condition for chitosan production. However, positive results were observed for asparagine with corn steep liquor with a high value in chitosan production.

### 2.3. Chitosan Characterization

**Figure 3:**
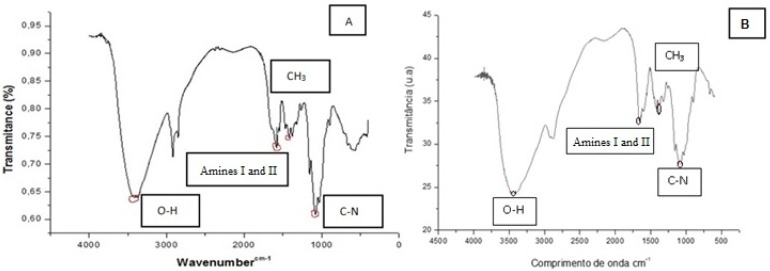
Infrared absorption spectra (**A**) Chitosan produced by microbiological *Cunninghamella elegans* grown on sucrose, asparagine and corn steep liquor medium (C:N; 2:6) and (**B**) Commercial chitosan.

**Figure 4:**
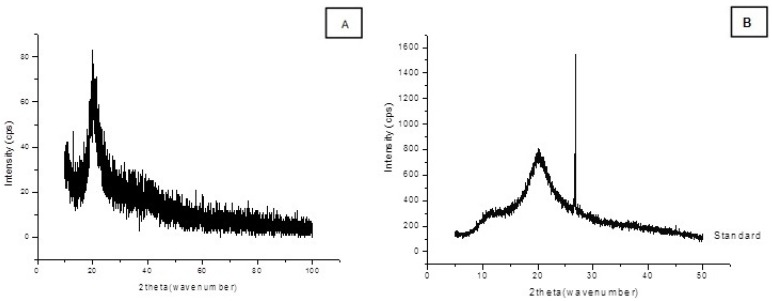
X-ray diffractograms of chitosan. (**A**) Microbiological chitosan of *C. elegans* in the best condition of the full factorial design. (**B**) X-ray diffractograms of commercial chitosan.

The physico-chemical characterization by X-ray diffraction and infrared absorption spectra of the isolated polymer are shown in Figure 3A, B and Figure 4A, B. In the infrared absorption spectrum, bands were observed related to OH-axial stretching between 3406 and 3432 cm^−1^, superimposed on the NH stretching band; with axial deformation of amide C=O at about 1639 cm^−1^, angular deformation of NH at approximately 1560 cm^−1^; axial deformation of amide-CN around 1421 cm^−1^; symmetrical angular deformation in CH_3_ 1379 cm^−1^; -CN axial deformation of amino groups between 1125–1250 cm^−1^ and polysaccharide structures bands in the region between 890–1150 cm^−1^. Therefore, the infrared absorption spectra profile of the chitosan isolated from *C. elegans* is supported by the literature [1,2,3,11,20]. The infrared spectroscopy of chitosan obtained by *C. elegans* at the central point of the 2³ factorial experimental design and commercial chitosan were used to determine the degree of deacetylation [35,41]. The degree of deacetylation of chitosan and the polymer microbial standard obtained were 75.25% and 78.5%, respectively, verifying through spectra in the infrared region that the degree of deacetylation of microbial chitosan exceeds the commercial standard by 11%. In an investigation of the use of chitosan for removing pollutants in textile effluent Lima e Silva [4] characterized the chitosan sample extracted from the mycelium of *C. elegans* and showed that the quality of the degree of deacetylation was slightly superior to the commercial standard used as a reference. In an experiment carried out to obtain chitosan produced by the filamentous fungus *Rhizopus arrhizus* observing a degree of deacetylation of 88.2%, similar statistical results to those in this study were found [20].

From the X-ray diffraction data obtained from chitosan which was obtained microbiologically from *C. elegans* under the best conditions of the factorial design, the crystallinity index (I_CR_) was calculated, the result being 60.92%, which is higher than the crystallinity index (56.94%) of the standard sample (Figure 4A, B). By X-ray diffraction analysis, observations were made on the degree of ordering of the polymer, which can be influenced by many factors, particularly, the processes of isolation and lyophilization, as per the literature [24,35]. Thus, the crystallinity indices are related to the physico-chemical properties of the polymer, changes to which can be reported, thus confirming the high degree of deacetylation of the chitosan [24]. The results of this study are consistent with those in the literature [24,35].

## 3. Experimental

### 3.1. Microorganism and Maintenance

*Cunninghamella elegans* strain UCP/WFCC 0542 was isolated from mangrove sediments of the Rio Formoso, Pernambuco state of Brazil, and belongs to the Culture Collection of the “Universidade Católica de Pernambuco” (UCP), located in the Research Center of Environmental Science—NPCIAMB/UNICAP. The Collection is registered in World Federation for Culture Collection (WFCC). The fungus is maintained on Potato Dextrose Agar (PDA) medium at 5 °C.

### 3.2. Chemicals and Corn Steep Liquor

The chemical substances asparagine and sucrose were obtained from Merck (São Paulo, Brazil). The corn steep liquor residue from corn was kindly provided by local industry and was used as the nitrogen source as per the 2^3^ factorial design.

### 3.3. Biomass Production by C. Elegans

Aliquots of 10^7^ cells/mL were transferred to Petri dishes (9.0 cm/diameter) containing PDA medium, and incubated for 24 h at 28 °C. After this period twenty disks with a diameter of 6mm were cut and used as inoculum in 250 mL Erlenmeyer flasks containing 100 mL of the production medium as per the 2³ factorial designs (Table 2). The flasks were incubated in an orbital shaker at 150 rpm, 28 °C for 96 h. At the end of cultivation, the mycelial mass of *C. elegans* was obtained by vacuum filtration, washed with cold sterile water, frozen and lyophilized. It was then kept in a desiccator until constant weight. The biomass was determined gravimetrically and expressed as g/L.

**Table 2:** Independent variables used in 2^3^ full factorial designs.

Independent Variable		Level	
	−1	0	+1
Asparagine % (w/v)	0	0.25	0.50
Sucrose % (w/v)	0.10	0.15	0.20
Corn steep liquor % (v/v)	0.30	0.45	0.60

### 3.4. Extraction of Chitosan

The biopolymer chitosan was extracted by alkali/acid treatment which consists of deproteinizing the biomass, lyophilized with the addition of a 1M sodium hydroxide solution submitted at 121 °C for 15 min, separating the alkali-insoluble fraction (AIF) after centrifugation (4,000 rpm/15 min) by vacuum filtration. After separation of the AIF, it was washed and the material was filtered in alternate stages with saline solution (0.85%) and ice-cold distilled water until pH 7.0. The residue obtained after this step was subjected to treatment with acetic acid (30 mL 2%/v/g 100 °C for 15 min) centrifuged (4,000 rpm/15 min) and filtered. The chitin was washed with cold distilled water and centrifuged (4,000 rpm) until pH 7.0. Then it was placed in a drying oven (30 °C/48 h.) The supernatant was alkalinized to pH 9.0, kept in a refrigerator for 24 h for precipitation and concentration of the chitosan. Having obtained chitosan by centrifugation (4,000 rpm/15 min), it was washed with ice-cold distilled water and saline solution to pH 7.0. The chitosan was dried in an oven at 30 °C for 24–48 h [25,41,42]. The chitosan concentration was expressed in g/L.

### 3.5. Determining Total Carbon and Nitrogen

From the cultivation medium of the experimental design which produced the highest biomass and chitosan, chemical analyzes were performed to measure the concentrations of organic carbon and total nitrogen consumed by *C. elegans* and the remainder in the culture. The percentage of carbon calaculated by the volumetric method was evaluated by potassium dichromate and titration with ferrous sulphate. This method consists of oxidizing organic carbon contained in a given volume of a sample with 0.1 N solution of potassium dichromate in an acidic medium, followed by titrating the excess dichromate solution with 0.05 N ammonium ferrous sulfate, using ferroín solution as an indicator [43]. To determine total nitrogen, the classical Kjeldahl method was used which comprises two steps: (1) digestion of the sample to convert organic nitrogen into the ammonium ion, and (2) determination of the digested N-NH_4_, after alkaline distillation. Ammonium sulfate resulting from the digestion is heated with a base, thus releasing ammonia (NH_3_), and the reaction can be represented by the equation: NH_4_ OH-H_2_ O ⇔ NH_3_. Ammonia is collected in an acidic solution, and was determined by colorimetry by titration using standard solution [44].

### 3.6. Determination of pH

After culture, the cell-free metabolic liquid was subjected to determination of pH by potentiometry. All experiments were performed in triplicate.

### 3.7. Physical-Chemistry Methods

*Infra-red spectroscopy–IRS*: Two milligrams (2 mg) of chitosan sample previously dried at 60 °C under reduced pressure were used and homogenized with 100 mg of potassium bromide (KBr). The potassium bromide discs prepared were dried for 24 h at 110 °C under pressure. The infrared ray spectra were obtained using a spectrophotometer Fourier transform (FTIR), Bruker IFS Mod. Potassium bromide discs were used as a reference. The intensity of the bands of maximum absorption was determined by the baseline method. The degree of deacetylation (DA%) was determined as per the methodology described by [15] on applying the following equation:

DD (%) = [100(Abs1655/Abs3450)]/1.33
(1)

*Crystallinity index*: The X-ray diffractograms of chitosan were obtained in the X-Ray Laboratory of the Physics Department – Federal University of Pernambuco—UFPE. The measurement was taken using SIEMENS Model 5000 D X-ray equipment, Cu Kα radiation with λ = 1.542 A0, in a scanning range between 4° and 50° with a rate of 0.02 min^−1^. The interplanar distance was determined by the half height width of the peak of greatest intensity (I_C_). The crystallinity index (I_CR_) was determined with the following equation:

Crystallinity index (%) = 100{[I(θc) − I(θa)]/I(θc)}
(2)

where I (θc) is the relative intensity of the crystalline (2θ = 20°) and I (θa) corresponds to amorphous regions (2θ = 12°) for chitosan.

### 3.8. Factorial design

The validation of the biopolymer chitosan production and checking the influence of the carbon source and nitrogen on the mycelial growth of *C. elegans* were undertaken using Pareto diagrams. The data obtained from the experiments were subjected to statistical analysis by STATISTICA software version 7.0 (StatSoft Inc., Tulsa, OK, USA). A 2^3^ full factorial design was carried out to analyze the main effects and interactions of varying concentrations of asparagine, sucrose and corn steep liquor on the response variable of biomass and chitosan production (Table 2).

## 4. Conclusions

Biomass and chitosan production by *C. elegans* using fermentation process was made possible under the conditions studied here and the main effects and interactions that were produced by the carbon and nitrogen sources present in the low cost medium formulation were identified. The corn steep liquor proved to be an excellent source of amino acids and the only independent variable that significantly influences the increase of the nitrogen source. Therefore, the product was formed at a Carbon:Nitrogen ratio of 2:6 in the medium (sucrose, asparagine, added corn steep liquor). The best conditions provided high chitosan production and physico-chemical characteristics that are important for a biopolymer that is promising for several applications.
